# Synthetic Cyclolipopeptides Selective against Microbial, Plant and Animal Cell Targets by Incorporation of D-Amino Acids or Histidine

**DOI:** 10.1371/journal.pone.0151639

**Published:** 2016-03-23

**Authors:** Sílvia Vilà, Esther Badosa, Emilio Montesinos, Marta Planas, Lidia Feliu

**Affiliations:** 1 LIPPSO, Department of Chemistry, University of Girona, Campus Montilivi, Girona, Spain; 2 Laboratory of Plant Pathology, Institute of Food and Agricultural Technology-CIDSAV-XaRTA, University of Girona, Campus Montilivi, Girona, Spain; Faculdade de Medicina da Universidade de Lisboa, PORTUGAL

## Abstract

Cyclolipopeptides derived from the antimicrobial peptide c(Lys-Lys-Leu-Lys-Lys-Phe-Lys-Lys-Leu-Gln) (**BPC194**) were prepared on solid-phase and screened against four plant pathogens. The incorporation at Lys^5^ of fatty acids of 4 to 9 carbon atoms led to active cyclolipopeptides. The influence on the antimicrobial activity of the Lys residue that is derivatized was also evaluated. In general, acylation of Lys^1^, Lys^2^ or Lys^5^ rendered the sequences with the highest activity. Incorporation of a D-amino acid maintained the antimicrobial activity while significantly reduced the hemolysis. Replacement of Phe with a His also yielded cyclolipopeptides with low hemolytic activity. Derivatives exhibiting low phytotoxicity in tobacco leaves were also found. Interestingly, sequences with or without significant activity against phytopathogenic bacteria and fungi, but with differential hemolysis and phytotoxicity were identified. Therefore, this study represents an approach to the development of bioactive peptides with selective activity against microbial, plant and animal cell targets. These selective cyclolipopeptides are candidates useful not only to combat plant pathogens but also to be applied in other fields.

## Introduction

In the last decades the use of antimicrobial peptides in plant protection has been widely studied due to their excellent biological properties. This family of peptides display a wide spectrum of activity against bacteria and fungi [1–3], selectivity towards microbial targets, and a low frequency in developing microbial resistance [4, 5]. Despite being structurally diverse, antimicrobial peptides have some features in common. They are typically linear or cyclic sequences, contain around 10–50 amino acids, are cationic and have up to 50% of hydrophobic residues, and are able to adopt an amphipathic structure. These structural characteristics govern the mechanism of action of these peptides, which mainly target the microbial membrane [6–9].

A subfamily of antimicrobial peptides includes native lipopeptides which mainly consist of a short (six to seven D- and L-amino acids) linear or cyclic peptide sequence, with either a net positive or a negative charge, and containing a lipid tail [10–15]. They differ on the length and composition of the fatty acid tail and on the number, type and configuration of the amino acids in the peptide moiety. Lipopeptides also show a wide range of biological activities, such as antimicrobial, cytotoxic and surfactant. The general mechanism described for lipopeptides is similar to that of other antimicrobial peptides. They also target the microbial membrane and mainly act by (i) inhibiting the synthesis of cell wall components, such as (1,3)-β-D-glucan or chitin, or (ii) inducing membrane lysis by forming non-specific channels/pores [15–20]. Since this mode of action seems not to involve a specific receptor, resistance to lipopeptides is generally rare [15, 21].

Moreover, the acyl chain present in cyclolipopeptides is an important determinant of their antimicrobial activity. It increases their affinity towards the membrane favoring their insertion into the hydrophobic core of the bilayer. The acyl chain also influences the organization of peptides and the conformation that they adopt when interacting with membranes [16, 21–23]. The addition of a lipophilic acyl chain to antimicrobial peptides is an effective approach to increase their membrane affinity, thus improving their activity. This has been observed for lipopeptides resulting from the acylation of the N-terminus of cationic antimicrobial peptides with fatty acids of 8 to 18 carbons [16, 19, 21, 22, 24–27]. Interestingly, acylation of low active de novo-designed diastereomeric Lys,Leu-containing dodecapeptides provided lipopeptides with antimicrobial activity [28, 29]. Similarly, a number of ultrashort lipopeptides consisting of four amino acids linked to fatty acids have been described to be active *in vitro* against a range of bacteria and fungi [30–32]. Lipopeptides have also been described to be active against plant pathogens. Makovitzki et al reported positively charged tetrapeptides linked to a fatty acid as potent inhibitors of plant pathogenic fungi and bacteria *in vitro* and also active in planta [33]. In addition, natural cyclic lipopeptides of the iturin, fengycin, surfactin and polymyxin families have well-recognized antimicrobial activity against phytopathogenic microorganisms [11, 14, 34–37].

We have recently reported the synthesis of cyclic lipopeptides derived from the antimicrobial cyclic decapeptide c(Lys-Lys-Leu-Lys-Lys-Phe-Lys-Lys-Leu-Gln) (**BPC194**) [38, 39]. In particular, **BPC194** was acylated at Lys^5^ with butyric acid, octanoic acid, lauric acid, 12-hydroxylauric acid or palmitic acid. The resulting cyclic lipopeptides were screened against phytopathogenic bacteria and fungi, and for cytotoxicity against red blood cells. We observed that the fatty acid length influences both the antimicrobial and hemolytic activities, and that the incorporation of a long hydrophobic chain was associated with a decreased antimicrobial activity and an increased hemolysis. By contrast, acylation with a butanoyl group led to **BPC500** which displayed a good balance between both activities.

Based on the previously described knowledge, it seems that synthetic cyclolipopeptides are suitable candidates to develop derivatives to find selective compounds against microbial, animal and plant cells targets. For example, it is obvious that in the field of the antimicrobial therapy in animals and plants, compounds should show minimized hemolytic or phytotoxic activities. However, compounds with no antimicrobial activity but being hemolytic or phytotoxic may find applications in biomedical or agricultural fields.

The aim of the present work was to develop synthetic cyclolipopeptides selective against microbial, plant and animal cell targets. Taking into account that the acylation of a peptide can modulate its activity, herein we decided to broaden the previous study by acylating **BPC194** with a range of fatty acids in order to find sequences with a wide spectrum of action. In particular, we evaluated the influence of the length and position of the fatty acid chain on the biological activity. Furthermore, taking into account that we have previously observed that the presence of a D-amino acid or of a His residue generally renders less hemolytic peptides [40–43], we decided to include in this study cyclolipopeptides incorporating a D-Lys, a D-Phe or a His. All peptides were screened against the plant pathogenic bacteria *Erwinia amylovora*, *Pseudomonas syringae* pv. *syringae* and *Xanthomonas axonopodis* pv. *vesicatoria*, and the plant pathogenic fungus *Fusarium oxysporum*. Hemolytic activity against human red blood cells and phytotoxicity in tobacco leaves were also determined.

## Materials and Methods

### Synthesis of cyclolipopeptides

Cyclolipopeptides were synthesized following the protocol previously described [39]. For further details concerning the synthesis of the derivatives incorporating a lipodipeptidyl tail see S1 File.

### Bacterial and fungal strains and growth conditions

The following plant pathogenic bacterial strains were used: *Erwinia amylovora* PMV6076 (Institut National de la Recherche Agronomique, Angers, France), *Pseudomonas syringae* pv. *syringae* EPS94 (Institut de Tecnologia Agroalimentària, Universitat de Girona, Spain) and *Xanthomonas axonopodis* pv. *vesicatoria* 2133–2 (Instituto Valenciano de Investigaciones Agrarias, Valencia, Spain). All bacteria were stored in Luria Bertani (LB) broth supplemented with glycerol (20%) and maintained at -80°C. Bacteria were scrapped from LB agar after growing for 24–48 h at 25°C. The cell material was suspended in sterile water to obtain a suspension of 10^8^ CFU mL^-1^.

*Fusarium oxysporum* f. sp. *lycopersici* FOL 3 race 2 (ATCC 201829, American Type Culture Collection) was used as fungal pathogen. The strain was cultured on potato dextrose agar (PDA) plates (Difco) and microconidia were obtained after growth at 25°C. The concentration of conidia was determined using a hemacytometer and adjusted to 10^4^ conidia mL^-1^.

### Antimicrobial activity

Lyophilized peptides were solubilized in sterile Milli-Q water to a final concentration of 1 mM and filter sterilized through a 0.22-μm pore filter. For minimum inhibitory concentration (MIC) assessment, dilutions of the compounds were made to obtain a stock concentration of 500, 250, 125, 62.5 and 31.1 μM. For antibacterial activity twenty microlitres of each dilution were mixed in a microtiter plate well with 20 μL of the corresponding suspension of the bacterial indicator, 160 μL of Trypticase Soy Broth (TSB) (BioMèrieux, France) to a total volume of 200 μL. For antifungal activity 20 μL of each stock solution were mixed in a microtiter plate well with 80 μL of the corresponding suspension of the fungal pathogen and 100 μL of double concentrated PDB to a total volume of 200 μL containing 0.003% w/v of choramphenicol.

Three replicates for each strain, compound and concentration were used. Microbial growth was determined by optical density measurement at 600 nm (Bioscreen C, Labsystem, Helsinki, Finland). For antibacterial activity microplates were incubated at 25°C with 20 sec shaking before hourly absorbance measurement for 48 h. For antifungal activity microplates were incubated at 20°C with 1 min shaking before absorbance measurement that were done every two hours during seven days. The experiment was repeated twice. The MIC was taken as the lowest compound concentration with no growth at the end of the experiment.

### Hemolytic activity

The hemolytic activity of the compounds was evaluated by determining hemoglobin release from erythrocyte suspensions of horse blood (5% vol/vol)(Oxoid) as previously described [44]. Blood was centrifuged at 6000g for 5 min, washed three times with TRIS buffer (10 mM TRIS, 150 mM NaCl, pH 7.2) and diluted.

Compounds were solubilized in TRIS buffer and mixed with horse erythrocytes and the final concentrations tested were 250 and 150 μM. The mixture was incubated under continuous shaking for 1 h at 37°C. Then, the tubes were centrifuged at 3500g for 10 min, 80 μL aliquots of the supernatant transferred to 100-well microplates (Bioscreen), diluted with 80 μL water, and the absorbance measured at 540 nm (Bioscreen). Complete hemolysis was obtained by the addition of melittin at 100 μM (Sigma-Aldrich Corporation, Madrid, Spain). The percentage of hemolysis (H) was calculated using the equation: H = 100×[(Op−Ob)/(Om−Ob)], where Op was the density for a given compound concentration, Ob for the buffer, and Om for the melittin positive control.

### Bactericidal activity

The bactericidal activity of peptides was determined for **BPC194** and the cyclolipopeptides **BPC500**, **BPC676**, **BPC686**, **BPC688**, **BPC714** and **BPC728**. In a total volume of 5 mL, 4 mL of Luria-Bertani (LB) broth were mixed with 0.5 ml of a suspension of *X*. *axonopodis* pv. *vesicatoria* adjusted to 10^8^ CFU mL^-1^ and 0.5 mL of the peptide solution at 50 μM (final concentration at 5 μM). Aliquots of 500 μL were removed at 30-min intervals during 3 h and the appropriate dilutions were spread onto LB agar using a spiral plater (Eddy Jet; IUL instruments, Spain). Triplicates were performed and colonies were counted after 48 h of incubation at 25°C with an automatic counter (Flash and Go; IUL instruments, Spain) by incorporating the software CounterMat version 5.0.

### Phytotoxicity

**BPC194** and a set of 24 cyclolipopeptides were evaluated for their phytotoxicity as described previously [44]. Peptide solutions of 50, 100, 150 and 250 μM were infiltrated (100 μL) into the mesophylls of fully expanded tobacco leaves. Six independent inoculations were carried out in a single leaf, and at least three independent inoculations were performed per peptide and concentration randomly distributed in diferent leaves and plants. Control infiltrations with water (negative control) or mellitin (positive control) at the same molar concentration were performed. The appearance of symptoms on the leaves was followed for 48 h after infiltration and measured as a lesion diameter.

## Results

### Design of the cyclolipopeptides

**Fig 1:**
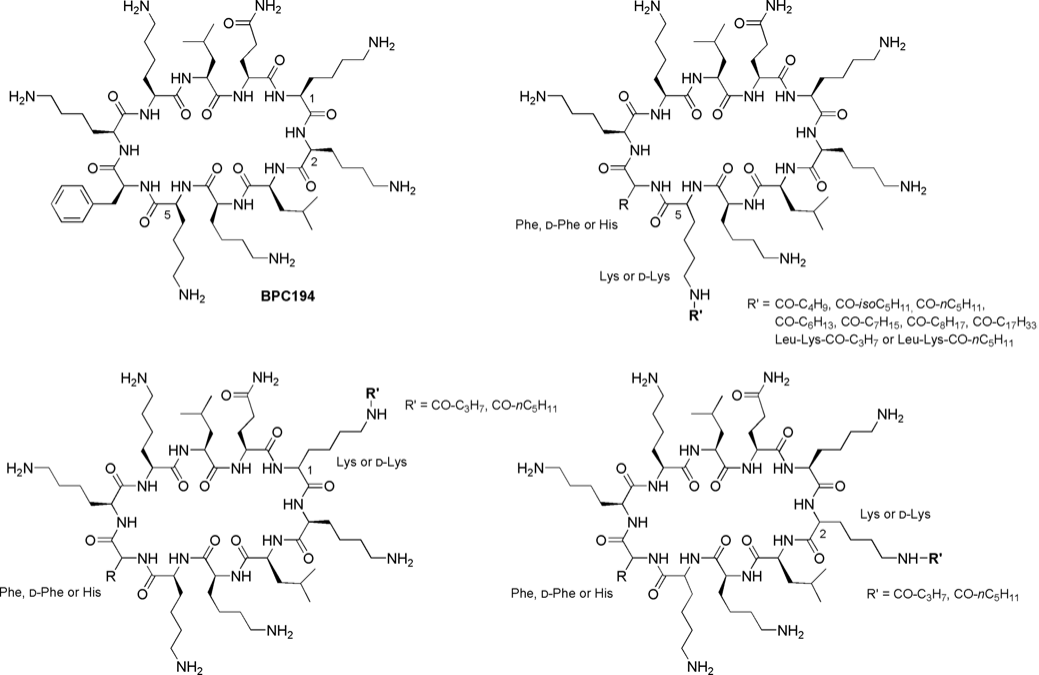
Structure of cyclolipopeptides derived from BPC194.

Cyclolipopeptides were designed based on the structure of the antimicrobial cyclic peptide c(Lys-Lys-Leu-Lys-Lys-Phe-Lys-Lys-Leu-Gln) (**BPC194**) [38] by incorporating a fatty acid chain at the *N*^*ε*^-amino group of a Lys residue (Fig 1 and Table 1). We evaluated the influence on the biological activity of the following issues: (i) the length of the fatty acid chain, (ii) the Lys residue that is derivatized, (iii) the incorporation of one or two D-amino acids, and (iv) the replacement of the Phe with a His.

**Table 1:** Sequences of the cyclolipopeptides.

Peptide	Structure^a^	Peptide	Structure^a^
*Parent peptide*			
**BPC194**	c(KKLKKFKKLQ)		
*Cyclolipopeptides with a different acyl chain at Lys*^*5*^			
**BPC498**	c(KKLKK(CO-C_7_H_15_)FKKLQ)	**BPC592**	c(KKLKK(CO-C_6_H_13_)FKKLQ)
**BPC500**	c(KKLKK(CO-C_3_H_7_)FKKLQ)	**BPC594**	c(KKLKK(CO-C_8_H_17_)FKKLQ)
**BPC526**	c(KKLKK(CO-C_4_H_9_)FKKLQ)	**BPC530**	c(KKLKK(CO-C_11_H_23_)FKKLQ)
**BPC504**	c(KKLKK(CO-*iso*C_5_H_11_)FKKLQ)	**BPC524**	c(KKLKK(CO-C_11_H_22_OH)FKKLQ)
**BPC528**	c(KKLKK(CO-*n*C_5_H_11_)FKKLQ)	**BPC502**	c(KKLKK(CO-C_15_H_31_)FKKLQ)
**BPC596**	c(KKLKK(LK-CO-*n*C_5_H_11_)FKKLQ)	**BPC622**	c(KKLKK(CO-C_17_H_33_)FKKLQ)
*Cyclolipopeptides with an acyl chain at a different Lys*			
**BPC582**	c(KKLKKFKK(CO-*n*C_5_H_11_)LQ)	**BPC708**	c(KK(CO-C_3_H_7_)LKKFKKLQ)
**BPC584**	c(KKLKKFK(CO-*n*C_5_H_11_)KLQ)	**BPC590**	c(K(CO-*n*C_5_H_11_)KLKKFKKLQ)
**BPC586**	c(KKLK(CO-*n*C_5_H_11_)KFKKLQ)	**BPC710**	c(K(CO-C_3_H_7_)KLKKFKKLQ)
**BPC588**	c(KK(CO-*n*C_5_H_11_)LKKFKKLQ)		
*Cyclolipopeptides with a D-Phe*			
**BPC712**	c(KKLKK(CO-C_3_H_7_)fKKLQ)	**BPC668**	c(KKLKK(CO-C_7_H_15_)fKKLQ)
**BPC726**	c(KKLKK(LK-CO-C_3_H_7_)fKKLQ)	**BPC714**	c(KK(CO-C_3_H_7_)LKKfKKLQ)
**BPC624**	c(KKLKK(CO-*n*C_5_H_11_)fKKLQ)	**BPC680**	c(KK(CO-*n*C_5_H_11_)LKKfKKLQ)
**BPC626**	c(KKLKK(CO-*iso*C_5_H_11_)fKKLQ)	**BPC716**	c(K(CO-C_3_H_7_)KLKKfKKLQ)
**BPC674**	c(KKLKK(LK-CO-*n*C_5_H_11_)fKKLQ)	**BPC686**	c(K(CO-*n*C_5_H_11_)KLKKfKKLQ)
*Cyclolipopeptides with a D-Lys*			
**BPC702**	c(KKLKk(CO-C_3_H_7_)FKKLQ)	**BPC666**	c(KKLKk(CO-C_7_H_15_)FKKLQ)
**BPC724**	c(KKLKk(LK-CO-C_3_H_7_)FKKLQ)	**BPC678**	c(Kk(CO-*n*C_5_H_11_)LKKFKKLQ)
**BPC628**	c(KKLKk(CO-*n*C_5_H_11_)FKKLQ)	**BPC704**	c(Kk(CO-C_3_H_7_)LKKFKKLQ)
**BPC630**	c(KKLKk(CO-*iso*C_5_H_11_)FKKLQ)	**BPC684**	c(k(CO-*n*C_5_H_11_)KLKKFKKLQ)
**BPC672**	c(KKLKk(LK-CO-*n*C_5_H_11_)FKKLQ)	**BPC706**	c(k(CO-C_3_H_7_)KLKKFKKLQ)
*Cyclolipopeptides with a D-Phe and a D-Lys*			
**BPC632**	c(KKLKk(CO-*n*C_5_H_11_)fKKLQ)	**BPC634**	c(KKLKk(CO-*iso*C_5_H_11_)fKKLQ)
*Cyclolipopeptides with a His*			
**BPC718**	c(KKLKK(CO-C_3_H_7_)HKKLQ)	**BPC670**	c(KKLKK(CO-C_7_H_15_)HKKLQ)
**BPC728**	c(KKLKK(LK-CO-C_3_H_7_)HKKLQ)	**BPC682**	c(KK(CO-*n*C_5_H_11_)LKKHKKLQ)
**BPC636**	c(KKLKK(CO-*n*C_5_H_11_)HKKLQ)	**BPC720**	c(KK(CO-C_3_H_7_)LKKHKKLQ)
**BPC638**	c(KKLKK(CO-*iso*C_5_H_11_)HKKLQ)	**BPC688**	c(K(CO-*n*C_5_H_11_)KLKKHKKLQ)
**BPC676**	c(KKLKK(LK-CO-*n*C_5_H_11_)HKKLQ)	**BPC722**	c(K(CO-C_3_H_7_)KLKKHKKLQ)

^a^ CO-C_3_H_7_, butanoyl; CO-C_4_H_9_, pentanoyl; CO-*n*C_5_H_11_, hexanoyl; CO-*iso*C_5_H_11_, 4-methylpentanoyl; CO-C_6_H_13_, 2-methylhexanoyl; CO-C_7_H_15_, octanoyl; CO-C_8_H_17_, 4-methyloctanoyl; CO-C_11_H_22_OH, 12-hydroxylauroyl; CO-C_11_H_23_, lauroyl; CO-C_15_H_31_, palmitoyl; CO-C_17_H_33_, oleoyl.

To address the first issue, Lys^5^ was derivatized with pentanoic acid (**BPC526**), 4-methylpentanoic acid (**BPC504**), hexanoic acid (**BPC528**), 2-methylhexanoic acid (**BPC592**), 4-methyloctanoic acid (**BPC594**) and oleic acid (**BPC622**) (Table 1). Taking into account that the structure of a variety of natural cyclolipopeptides usually contains a lipopeptidyl tail [11, 12], Lys^5^ was also derivatized with the dipeptide Leu-Lys bearing a hexanoyl chain (**BPC596**). Concerning the second issue, each Lys residue of **BPC194** was derivatized with hexanoic acid and Lys^1^ and Lys^2^ also with butyric acid.

Regarding the incorporation of D-amino acids, the Phe and/or the Lys bearing the fatty acid chain were replaced with their D-enantiomer (Table 1). Derivatives incorporating a His instead of the Phe were also considered. In all these sequences the Lys that was acylated was that at position 1, 2 or 5. The fatty acids employed for the acylation at position 5 were butyric acid, hexanoic acid, 4-methylpentanoic acid and octanoic acid, whereas butyric acid and hexanoic acid were used at positions 1 or 2. Moreover, the side-chain of Lys^5^ was elongated with the dipeptide Leu-Lys bearing a butanoyl or a hexanoyl chain.

### Solid-phase synthesis of the cyclolipopeptides

**Fig 2:**
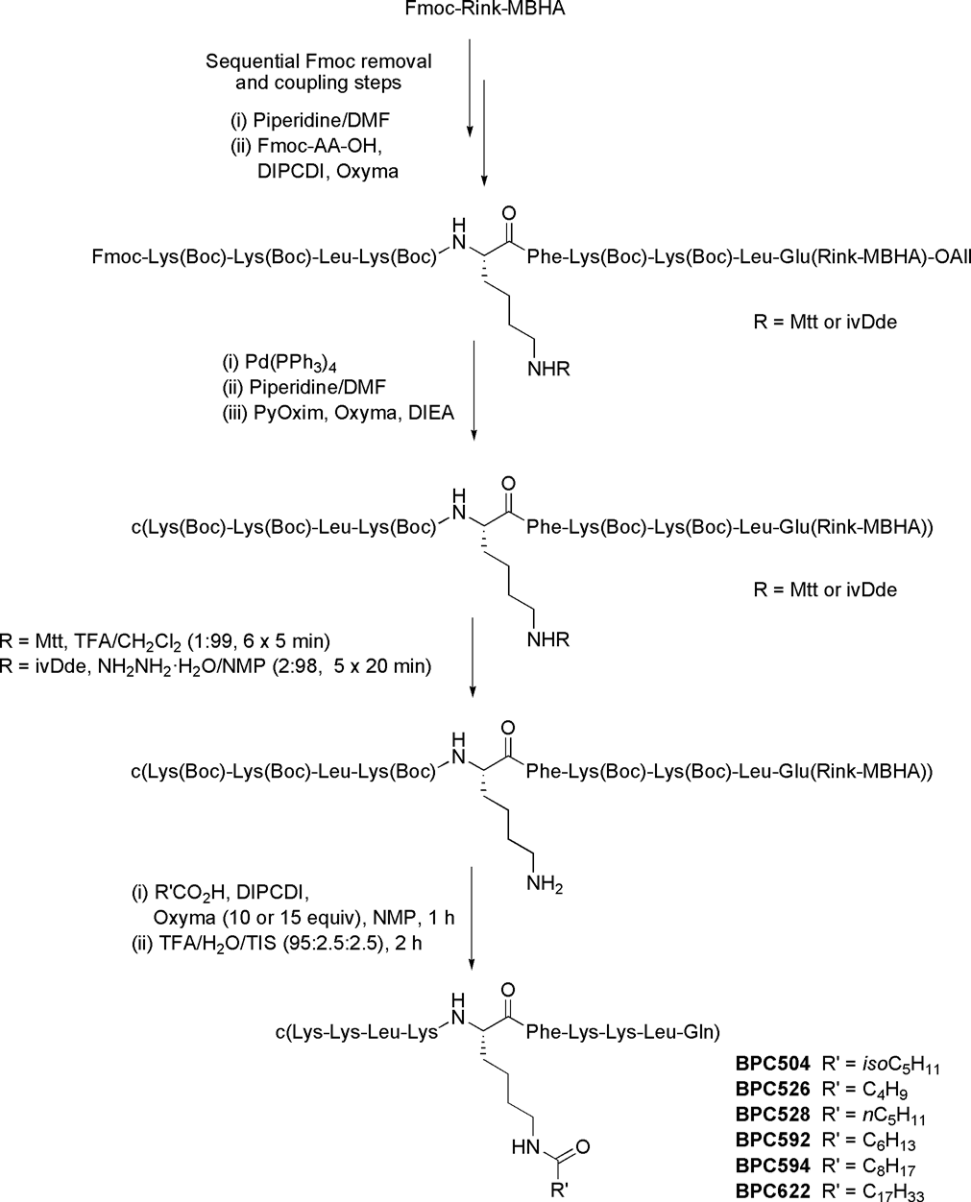
General strategy for the synthesis of cyclolipopeptides

Cyclolipopeptides were synthesized following a standard three-dimensional orthogonal 9-fluorenylmethoxycarbonyl (Fmoc)/*tert*-butyl (*t*Bu)/allyl (All) strategy that involved the synthesis of the corresponding linear peptidyl resin followed by solid-phase cyclization and acylation (See ref 39 and S1 File for further details). The general synthetic protocol is depicted in Fig 2 for compounds bearing the acyl chain at position 5. Cyclolipopeptides were analyzed by HPLC and characterized by ESI/MS and HRMS. They were obtained in purities ranging from 86–99% (Tables A and B in S1 File).

### Antimicrobial activity

**Fig 3:**
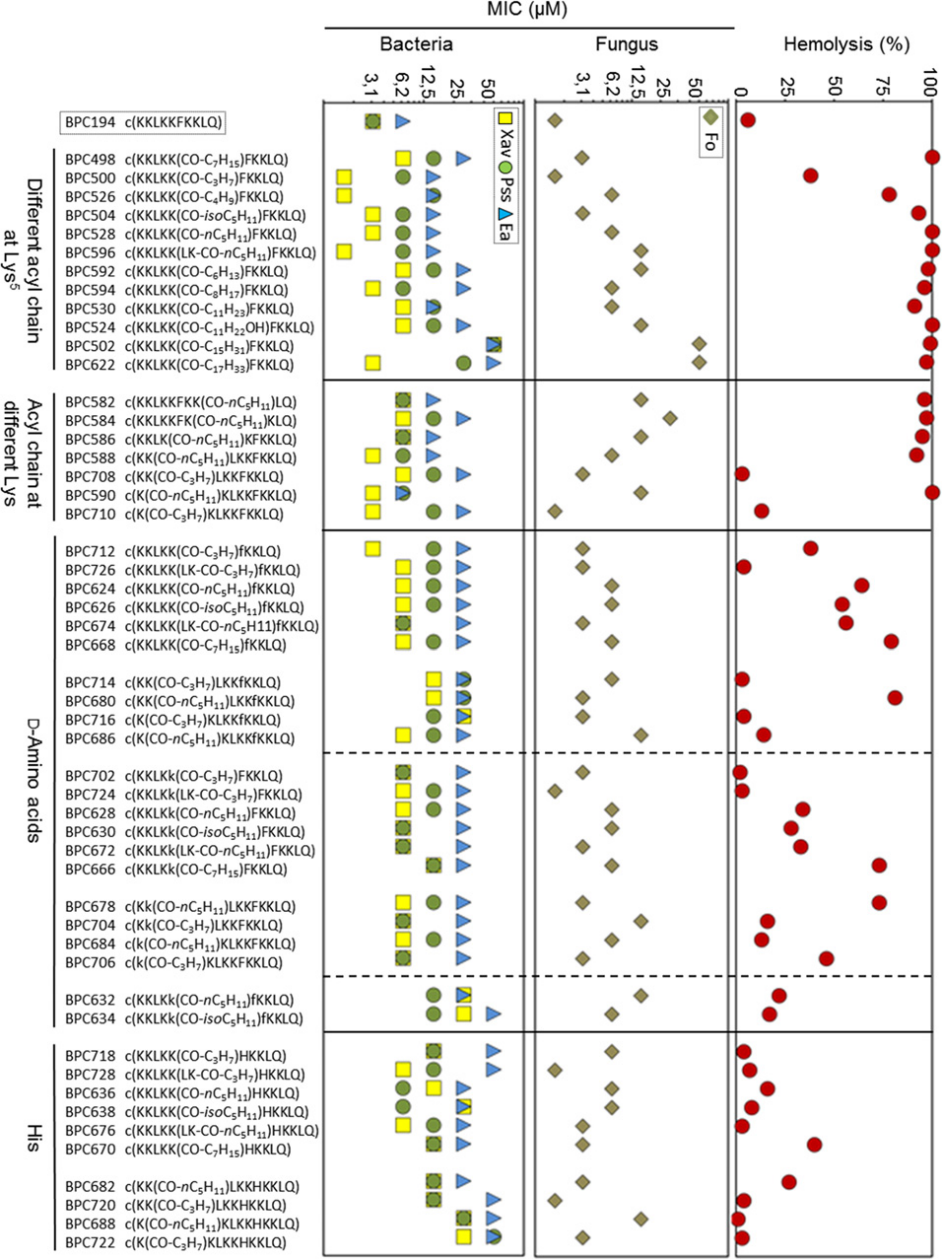
Antimicrobial and hemolytic activities of cyclolipopeptides. Sequences of the cyclolipopeptides grouped according to their structure are depicted. Antimicrobial activity is given as the minimal concentration that inhibits growth (MIC). The MIC axis is in logarithmic scale and for each sequence the lowest values of the MIC range are represented. The hemolytic activity was measured at 250 μM and is expressed as a percentage compared to melittin as a standard. Data can be found in Tables C and D in S1 File.

Cyclolipopeptides were tested for *in vitro* growth inhibition of *E*. *amylovora*, *P*. *syringae* pv. *syringae* and *X*. *axonopodis* pv. *vesicatoria*, and *F*. *oxysporum* at 3.1, 6.2, 12.5, 25, and 50 μM (Fig 3, Tables C and D in S1 File). Cyclic peptide **BPC194** and cyclolipopeptides incorporating an octanoyl (**BPC498**), butanoyl (**BPC500**), palmitoyl (**BPC502**), 12-hydroxylauroyl (**BPC524**) or lauroyl (**BPC530**) chain were also included for comparison purposes [39]. Except for **BPC502**, all peptides were active against at least one pathogen with MIC <25 μM. Notably, 40 compounds out of 51 showed activity against three pathogens with MIC <25 μM. *X*. *axonopodis* pv. *vesicatoria* and *F*. *oxysporum* were the most sensitive pathogens to these compounds.

Among the set of 12 cyclolipopeptides bearing a fatty acid chain at Lys^5^, three sequences were more active than **BPC194** against *X*. *axonopodis* pv. *vesicatoria* (MIC < 3.1 μM) and four were as active as **BPC194** (MIC of 3.1 to 6.2 μM). Against *P*. *syringae* pv. *syringae* and *E*. *amylovora*, five and six sequences, respectively, were slightly less active (MIC of 6.2 to 12.5 and MIC of 12.5 to 25 μM, respectively) than the parent peptide. Regarding the antifungal activity, the cyclolipopeptides of this set were less active than **BPC194** against *F*. *oxysporum*, but one sequence was as active as **BPC194** (MIC <3.1 μM) and six sequences displayed MIC <12.5 μM. Among this set, sequences bearing a butanoyl (**BPC500**), a 4-methylpentanoyl (**BPC504**), a hexanoyl chain (**BPC528**) or a 4-methyloctanoyl (**BPC594**) were the most active with MIC of <12.5 μM against three pathogens.

The analysis of the biological activity of cyclolipopeptides bearing a hexanoyl chain at the different Lys residues revealed that derivatization of Lys^1^, Lys^2^ or Lys^5^ yielded peptides **BPC590**, **BPC588** and **BPC528** respectively, with MIC values of 3.1 to 25 μM. Acylation of these residues with butyric acid also led to active peptides (**BPC500**, **BPC708** and **BPC710**).

The incorporation of one or two D-amino acids led to peptides with low activity against *E*. *amylovora* (MIC of 25 to 50 μM) and with important activity against the other three pathogens. Sixteen, six and nineteen cyclolipopeptides out of twenty-two displayed MIC <12.5 μM against *X*. *axonopodis* pv. *vesicatoria*, *P*. *syringae* pv. *syringae*, and *F*. *oxysporum*, respectively. In particular, the replacement of the L-Phe with its D-enantiomer generally decreased the antibacterial activity. In contrast, the presence of a D-Phe rendered peptides active against *F*. *oxysporum* with MIC <12.5 μM. Cyclolipopeptides incorporating a D-Lys where generally more active than those containing a D-Phe, especially the activity increased against *P*. *syringae* pv. *syringae*. The presence of a D-Lys and a D-Phe yielded low active peptides (**BPC632** and **BPC634**). Best peptides with a D-amino acid were **BPC672**, **BPC674**, **BPC702**, **BPC706**, and **BPC712** with MIC of 3.1 to 12.5 μM against *X*. *axonopodis* pv. *vesicatoria*, MIC of 6.2 to 12.5 μM against *P*. *syringae* pv. *syringae*, and MIC of 3.1 to 6.2 μM against *F*. *oxysporum*. Finally, when Phe was replaced with a His cyclolipopeptides were less active against the bacteria tested, but they were slightly more active against *F*. *oxysporum*.

**Fig 4:**
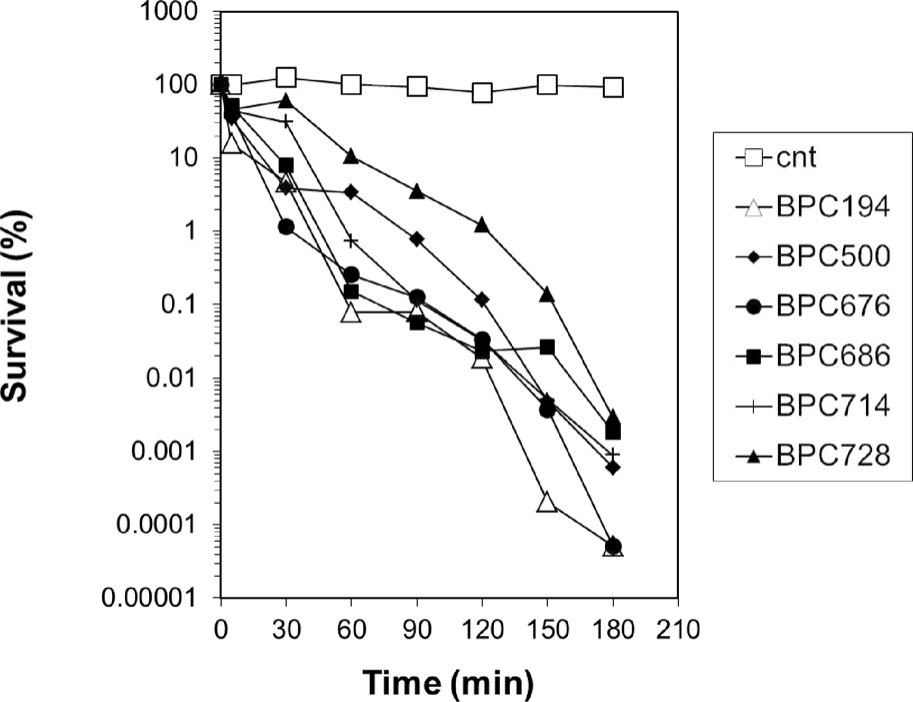
Kinetics of survival of *X*. *axonopodis* pv. *vesicatoria* in the presence of BPC194 (△) or selected cyclolipopeptides. Bacterial suspensions were untreated (□) or treated with 5 μM concentrations of **BPC500** (⧫), **BPC676** (●), **BPC686** (■), **BPC714** (+) and **BPC728** (π). Viable cells were determined at different time intervals.

The bactericidal activity (killing assay) of five representative peptides, **BPC500**, **BPC676**, **BPC686**, **BPC714** and **BPC728**, was determined by comparing the time course to kill mid-logarithmic-phase culture suspensions of *X*. *axonopodis* pv. *vesicatoria* (Fig 4). At the concentration tested (5 μM), all peptides exhibited a bactericidal activity with a similar behaviour to that of the parent peptide **BPC194**.

### Hemolytic activity

The toxicity of cyclolipopeptides to eukaryotic cells was determined as the ability to lyse erythrocytes in comparison to melittin. Percent hemolysis at 250 μM is shown in Fig 3 and in Tables C and D in S1 File.

Cyclolipopeptides bearing a fatty acid at Lys^5^ showed a hemolytic activity between 38 and 100% at this concentration. The incorporation of a hexanoyl chain at each of the other Lys residues also led to peptides with high hemolysis (92–100%). By contrast, acylation of Lys^1^ and Lys^2^ with butyric acid afforded non hemolytic cyclolipopeptides (13 and 3%, respectively). In general, cyclolipopeptides bearing one or two D-amino acids were less hemolytic than the L-counterparts. In particular, ten out of twenty-two sequences displayed a hemolysis <25% at 250 μM and six peptides, a hemolysis ranging from 25 to 50%. Moreover, cyclolipopeptides with a D-Lys were less hemolytic than those with a D-Phe. The incorporation of a His residue rendered the least hemolytic peptides with a percent hemolysis between 1 and 40% at 250 μM.

### Phytotoxicity

**Fig 5:**
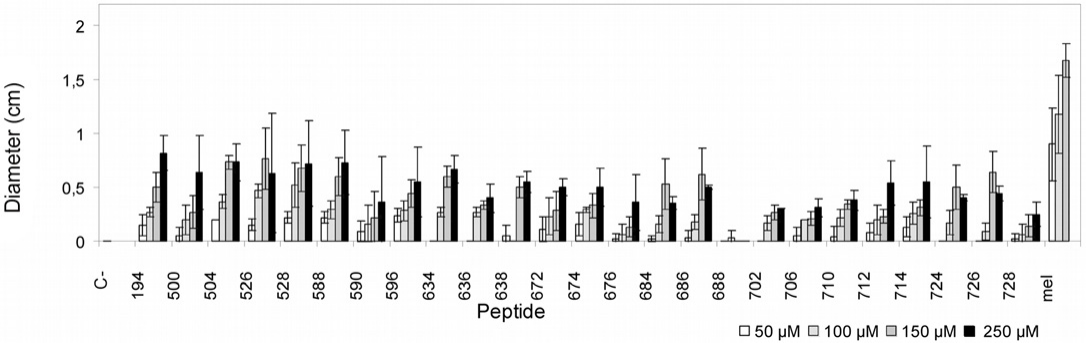
Phytotoxicity of BPC194 and a set of cyclolipopeptides. Phytotoxicity was determined at 50, 100, 150, and 250 μM, as the size of the lesions in infiltrated tobacco leaves. Phytotoxicity was compared to melittin. Vertical bars within each column indicate confidence interval at the mean.

The toxicity of a set of 24 cyclolipopeptides in tobacco leaves was assessed by infiltrating 100 μL of a 50, 100, 150 and 250 μM solution of each compound into the mesophylls of the leaves (Fig 5). **BPC194** was also included for comparison purposes, and as a reference control, the nonspecific and nonselective toxic peptide mellitin was used. After 48 h of infiltration, a brown necrotic area of around 2 cm diameter was observed for mellitin peptide at 250 μM. In contrast, **BPC194** and cyclolipopeptides were significantly less phytotoxic causing a necrosis between 0–0.82 cm at this concentration. A dose-response effect was observed with a clear phytotoxicity increase from 50 to 250 μM. The most phytotoxic peptides were **BPC194**, **BPC504**, **BPC528** and **BPC588** for which the size of the lesions were between 0.72–0.82 cm. Cyclolipopeptides **BPC590**, **BPC676**, **BPC684**, **BPC688**, **BPC702**, **BPC706**, **BPC710** and **BPC728** were the least phytotoxic producing a necrosis between 0–0.38 cm.

## Discussion

There is currently great interest to develop functional peptides selective against microbial, animal or plant cells targets. In the present work we demonstrated that cyclolipopeptides offer great expectations for developing a wide range of selective functional peptides.

Previous reports on lipopeptides have shown that both the composition of the peptide moiety and the type of the lipophilic part are sensitive to modification [19]. This modification may increase peptide activity or change its specificity [1, 15, 20]. Studies analyzing the influence of the hydrophobic chain have been carried out on linear as well as on cyclic peptide sequences [12, 21]. In particular, acylated D,L-amino acid-containing antimicrobial dodecapeptides showed the optimal antibacterial activity for acyl chains of 10 and 12 carbons whereas the highest antifungal activity was observed for longer acyl chains containing 14 and 16 carbons [15, 29]. A similar trend for antibacterial activity has been reported for lipopeptides derived from cathepsin G 117–136, where N-terminal attachment of fatty acids having C-8, C-10 and C-12 hydrocarbon chains, but not C-2, C-4, C-6, C-14, C-16 or C-18, increased significantly the bactericidal activity against many Gram-positive bacteria [45]. Other studies have focused on D,L-amino acid containing tetrapeptides and have demonstrated that acylation with fatty acids of 12, 14, and 16 carbons endowed them with antimicrobial activity [30, 32]. However, neither the hydrophobicity of the peptide chain nor the length of the aliphatic chain correlated with the biological activity of the lipopeptides [30].

Our recent studies on the antimicrobial activity of cyclolipopeptides showed that the length of the hydrophobic chain is also a key determinant of the activity but, unlike the above reports, the presence of a long hydrophobic chain is related to a decrease of the activity [39]. The results reported in the present work are in agreement with these preliminary observations. Thus, cyclolipopeptides with a palmitoyl (**BPC502**) and an oleoyl (**BPC622**) chain were not active. In contrast, cyclolipopeptides with acyl substituents of 4 to 9 carbon atoms displayed significant activity against the four pathogens. Moreover, the acylation with hexanoic acid of the dipeptide chain linked to the *N*^ε^-amino group of Lys^5^ also afforded a sequence with important antimicrobial activity (**BPC596**). We also observed that the position of the hydrophobic chain influenced the activity of these cyclolipopeptides. For the hexanoyl group, the highest activity was obtained upon acylation of Lys^1^, Lys^2^ or Lys^5^ (**BPC590**, **BPC588**, and **BPC528**, respectively). Derivatization of Lys^1^ or Lys^5^ with a butanoyl chain (**BPC710** and **BPC500**, respectively) also rendered sequences with significant activity. Regarding the antifungal activity, no direct correlation was found between the length of the fatty acid and the MIC values against *F*. *oxysporum*. Moreover, cyclolipopeptides bearing an oleoyl or a palmitoyl chain did not display antifungal activity. In contrast, other authors described that lipopeptides gain potent antifungal activity being those with long aliphatic chains markedly active against fungi [16, 29, 30, 32, 46].

One important drawback of most antimicrobial lipopeptides is their hemolytic activity. Inclusion of D-amino acids into antimicrobial peptide sequences has been reported as a means to decrease the hemolytic activity while maintaining the antimicrobial activity [41]. Similarly, the hemolysis of this family of cyclolipopeptides was reduced by substituting the Phe or the Lys residue bearing the hydrophobic chain by the corresponding D-enantiomer. The lowest hemolytic cyclolipopeptides were those containing an acylated D-Lys residue. Interestingly, compared to their L-counterparts, the antimicrobial activity of peptides containing a D-amino acid was maintained. In particular, acyl substituents of 4 to 8 carbon atoms conferred them high activity, being cyclolipopeptides incorporating an acylated D-Lys more active than those containing a D-Phe.

Previous studies on antimicrobial linear undecapeptides also showed that replacement of a Phe by a His residue yielded peptides with low hemolysis, which was attributed to the higher hydrophilicity of the imidazole ring of His compared to the benzene ring of Phe, and with high antifungal activity against *F*. *oxysporum* [42]. For cyclolipopeptides, replacement of Phe by His afforded the least hemolytic sequences. Moreover, whereas the antibacterial activity decreased, the antifungal activity of these derivatives was maintained or improved.

Importantly, in general, cyclolipopeptides bearing a D-amino acid or a His were not hemolytic or displayed low hemolysis at concentrations up to 250 μM, which is 10 to 80 fold higher than the observed MIC values. Other studies claimed that lipopeptides were not hemolytic, but they were tested at much lower concentrations (20 or 50 μM or at the MIC values) than in the present work [16, 28, 30, 46].

Cyclolipopeptides did not cause significant lesions in tobacco leaves at concentrations up to 20 to 80 fold higher than the MIC and were considerably less phytotoxic than melittin. These results are in agreement with reports on short positively charged lipopeptides that are highly potent inhibitors of plant phytopathogens, but do not harm plant tissues when applied directly at the infection area [33]. Interestingly, in the present work cyclolipopeptides less phytotoxic than the parent peptide **BPC194** were identified.

**Fig 6:**
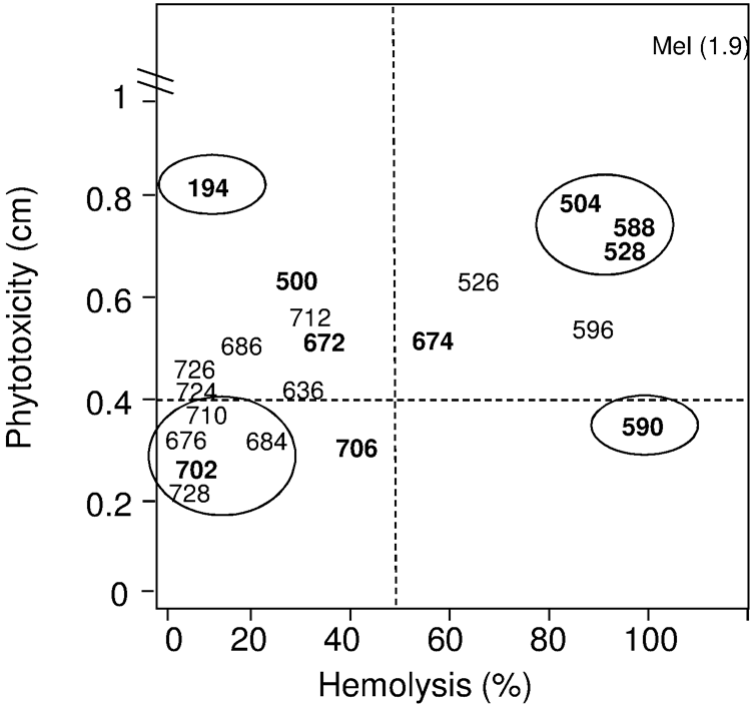
Relationship between phytotoxicity and hemolysis at 250 μM of cyclolipopeptides derived from BPC194 active against three pathogens with MIC <25 μM. Peptides in bold display MIC <12.5 μM against three pathogens.

Taking into account all the results of the present work, we demonstrated that starting from a cyclic lead peptide (**BPC194**) with high antimicrobial activity, low hemolysis and high phytotoxicity we were able to develop cyclolipopeptides with a differential biological activity profile (Fig 6). Thus, we identified a peptide highly hemolytic and poorly phytotoxic (**BPC590**), sequences with both high hemolytic activity and phytotoxicity (**BPC504**, **BPC528** and **BPC588**), and cyclolipopeptides neither hemolytic nor phytotoxic (**BPC676**, **BPC684**, **BPC702**, **BPC710** and **BPC728**). From the latter group, **BPC702** incorporating D-Lys^5^ acylated with a butanoyl group, was active against *X*. *axonopodis* pv. *vesicatoria*, *P*. *syringae* pv. *syringae* and *F*. *oxysporum* with MIC <12.5 μM, and showed no hemolysis and the lowest necrosis levels in tobacco leaves at 250 μM. These results also confirm previous data on how subtle changes in this family of peptides influence the biological activity [38], which difficults the prediction of the whole biological activity profile for a given peptide sequence.

In summary, we show herein that the combination of a peptidyl scaffold with inherent antimicrobial activity and an acyl substituent represents an approach to the development of potent cyclic peptides with selective activity against microbial, plant and animal cell targets. In particular, we identified cyclolipopeptides derived from the antimicrobial peptide **BPC194** with significant activity against phytopathogenic bacteria and fungi, but with differential hemolysis and phytotoxicity. As a general trend, the incorporation of a D-amino acid or a His led to sequences with a reduced hemolytic activity. Thus, this work paves the way to develop a promising group of lipopeptide candidates selective against microbial, animal or plant cells targets, useful not only in agriculture but also in other fields.

## Supporting Information

S1 File Table A in S1 File. Sequences, retention times and purities on HPLC, and mass spectrometry data of cyclolipopeptides. Table B in S1 File. Sequences, retention times and purities on HPLC, and mass spectrometry data of cyclolipopeptides. Table C in S1 File. Antimicrobial (MIC) and hemolytic activities of cyclolipopeptides containing all L-amino acids. Table D in S1 File. Antimicrobial (MIC) and hemolytic activities of cyclolipopeptides containing D-amino acids or a histidine residue.
(DOC)
