# Supplementary material for: Synthetic Cyclolipopeptides Selective against Microbial, Plant and Animal Cell Targets by Incorporation of D-Amino Acids or Histidine
Source: PLoS One. 2016 Mar 23;11(3):e0151639. doi: 10.1371/journal.pone.0151639 (PMC4805166; doi:10.1371/journal.pone.0151639)
Supplement: S1 File — Table A in S1 File. Sequences, retention times and purities on HPLC, and mass spectrometry data of cyclolipopeptides. Table B in S1 File. Sequences, retention times and purities on HPLC, and mass spectrometry data of cyclolipopeptides. Table C in S1 File. Antimicrobial (MIC) and hemolytic activities of cyclolipopeptides containing all L-amino acids. Table D in S1 File. Antimicrobial (MIC) and hemolytic activities of cyclolipopeptides containing D-amino acids or a histidine residue. (DOC) [file pone.0151639.s001.doc]

Supporting Information

Synthetic cyclolipopeptides selective against microbial, plant and animal cell targets by incorporation of D-amino acids or histidine

Sílvia Vilà, Esther Badosa, Emilio Montesinos*, Marta Planas*, and Lidia Feliu*

[*emilio.montesinos@udg.edu*](mailto:emilio.montesinos@udg.edu), [*marta.planas@udg.edu*](mailto:marta.planas@udg.edu), [*lidia.feliu@udg.edu*](mailto:lidia.feliu@udg.edu)

**Materials and methods**

General

Commercially available reagents were used throughout without purification. Solvents were purified and dried by passing them through an activated alumina purification system (MBraun SPS-800) or by conventional distillation techniques.

Flash chromatography purifications were performed on C_18_-reversed phase silica gel 100 not endcapped (230-400 mesh, Fluka).

All compounds were analyzed under standard analytical high-performance liquid chromatography (HPLC) conditions with a Dionex liquid chromatography instrument. Detection was performed at 220 nm. Analysis was carried out using a Kromasil 100 C_18_ (40 mm×4.6 mm, 3.5 μm) column with a 2-100% B linear gradient over 7 min at a flow rate of 1 mL/min. Solvent A was 0.1% aqueous trifluoroacetic acid (TFA), and solvent B was 0.1% TFA in CH_3_CN.

ESI-MS analyses were performed with an Esquire 6000 ESI ion Trap LC/MS (Bruker Daltonics) instrument equipped with an electrospray ion source (Serveis Tècnics de Recerca, University of Girona). The instrument was operated in the positive ESI(+) ion mode. Samples (5 μL) were introduced into the mass spectrometer ion source directly through an HPLC autosampler. The mobile phase (80:20 CH_3_CN/H_2_O at a flow rate of 100 μL/min) was delivered by a 1100 Series HPLC pump (Agilent). Nitrogen was employed as both the drying and nebulizing gas. HRMS were recorded under conditions of ESI with a Bruker MicroTof-Q IITM instrument using a hybrid quadrupole time-of-flight mass spectrometer (Serveis Tècnics de Recerca, University of Girona). Samples were introduced into the mass spectrometer ion source by direct infusion through a syringe pump and were externally calibrated using sodium formate. The instrument was operated in the positive ESI(+) ion mode.

Synthesis of linear peptidyl resins

The linear peptidyl resins were synthesized manually by the solid-phase method using standard Fmoc chemistry. Fmoc-Rink-MBHA resin (0.4 mmol/g) was used as solid support. Fmoc-Leu-OH, Fmoc-Lys(Boc)-OH, Fmoc-Lys(Mtt)-OH, Fmoc-Lys(ivDde)-OH, Fmoc-D-Lys(ivDde)-OH, Fmoc-Phe-OH, Fmoc-D-Phe-OH, Fmoc-His(Tr)-OH or Fmoc-Glu-OAll were used as amino acid derivatives. Fmoc group removal was achieved with piperidine/DMF (3:7, 2+10 min). Couplings of the Fmoc-amino acids (4 equiv) were mediated by ethyl 2-cyano-2-(hydroxyimino)acetate (Oxyma) (4 equiv), *N,N*’-diisopropylcarbodiimide (DIPCDI) (4 equiv) in DMF at room temperature for 1 h. The completion of the reactions was checked by the Kaiser test [1]. After each deprotection and coupling step, the resin was washed with DMF (6×1 min), and CH_2_Cl_2_ (3×1 min), and air dried. An aliquot of each resulting peptidyl resin was treated with TFA/H_2_O/triisopropylsilane (TIS) (95:2.5:2.5) for 2 h. Following TFA evaporation and diethyl ether extraction, the crude peptides were dissolved in H_2_O, lyophilized and analyzed by HPLC.

Synthesis of cyclic peptidyl resins

Cyclic peptidyl resins were prepared from the corresponding linear peptidyl resins. The C-terminal allyl ester of the linear peptidyl resin was cleaved by treatment with Pd(PPh_3_)_4_ (5 equiv) in CHCl_3_/AcOH/*N*-methylmorpholine (NMM) (3:2:1) under nitrogen for 3 h, and the resins were washed with tetrahydrofuran (3×2 min), NMP (3×2 min), DIEA/CH_2_Cl_2_ (1:19, 3×2 min), sodium *N,N*-diethyldithiocarbamate (0.03 M in NMP, 3×15 min), NMP (10×1 min) and CH_2_Cl_2_ (3×2 min). Fmoc was removed with piperidine/DMF (3:7, 2+10 min) followed by washes with DMF (6×1 min) and CH_2_Cl_2_ (3×1 min). Cyclization was carried out by treating the resulting resin with [ethyl cyano(hydroxyimino)acetato-*O*^2^]tri-1-pyrrolidinylphosphonium hexafluorophosphate (PyOxim) (5 equiv), Oxyma (5 equiv), and DIEA (10 equiv) in NMP under stirring for 24 h. Following washes with NMP (6×1 min) and CH_2_Cl_2_ (3×1 min), an aliquot of each cyclic peptidyl resin was cleaved by treatment with TFA/H_2_O/TIS (95:2.5:2.5) for 2 h. Following TFA evaporation and diethyl ether extraction, the crude peptides were dissolved in H_2_O, lyophilized, analysed by HPLC and characterized by mass spectrometry.

Synthesis of cyclolipopeptides

When starting from a cyclic peptidyl resin containing a Lys(Mtt) residue, the resin was first treated with TFA/CH_2_Cl_2_ (1:99, 6×5 min). The resulting resin was washed with CH_2_Cl_2_ (6×1 min), NMP (6×1 min), CH_2_Cl_2_ (6×1 min), and DIEA/NMP (5:95, 3×2 min). When starting from a cyclic peptidyl resin containing a Lys(ivDde) residue, the resin was first treated with NH_2_NH_2_·H_2_O/NMP (2:98, 5×20 min) under stirring. The resulting resin was washed with NMP (2×1 min), CH_2_Cl_2_ (2×1 min), MeOH (2×1 min), and NMP (2×1 min). Then, the resin was acylated by treatment with butyric acid, 4-methylpentanoic acid, pentanoic acid, hexanoic acid, 2-methylhexanoic acid, 4-methyloctanoic acid or octanoic acid (10 equiv), DIPCDI (10 equiv) and Oxyma (10 equiv) in NMP for 1 h. Incorporation of the oleoyl group was performed by treating the resin with oleic acid (15 equiv), DIPCDI (15 equiv) and Oxyma (15 equiv) in NMP for 1 h. Synthesis of the derivatives incorporating a lipodipeptidyl tail was carried out through subsequent coupling of Fmoc-Leu-OH and Fmoc-Lys(Boc)-OH followed by Fmoc removal and acylation with butyric acid or hexanoic acid. The resulting cyclolipopeptidyl resins were washed with NMP (6×1 min) and CH_2_Cl_2_ (6×1 min) and air dried. Completion of the reactions was checked with the Kaiser test [1]. Each resulting peptidyl resin was treated with TFA/H_2_O/TIS (95:2.5:2.5) for 2 h. Following TFA evaporation and diethyl ether extraction, the crude cyclic lipopeptides were dissolved in H_2_O and lyophilized. Cyclic lipopeptides were analyzed by HPLC and characterized by mass spectrometry.

[1] Kaiser E, Colescott RL, Bossinger CD, Cook P. Color test for detection of free terminal amino groups in the solid-phase synthesis of peptides. Anal Biochem. 1970;34: 595-598.

**TableA. Sequences, retention times and purities on HPLC, and mass spectrometry data of cyclolipopeptides**

| Peptide | Structure^a^ | *t*_R_ (min)^b^ | Purity (%)^c^ |  | | HRMS (ESI) | | |  |
| --- | --- | --- | --- | --- | --- | --- | --- | --- | --- |
|  |  |  |  | |  | | Calculated for | Found | |
| **BPC498** | c(KKLKK(CO-C_7_H_15_)FKKLQ) | 7.12 | 87 | |  | | 698.9920 [M+2H]^2+^ | 698.9919 | |
| **BPC500** | c(KKLKK(CO-C_3_H_7_)FKKLQ) | 6.53 | 96 | |  | | 670.9607 [M+2H]^2+^ | 670.9596 | |
| **BPC526** | c(KKLKK(CO-C_4_H_9_)FKKLQ) | 6.80 | 97 | |  | | 677.9685 [M+2H]^2+^ | 677.9699 | |
| **BPC504** | c(KKLKK(CO-*iso*C_5_H_11_)FKKLQ) | 6.96 | 87 | |  | | 684.9763 [M+2H]^2+^ | 684.9786 | |
| **BPC528** | c(KKLKK(CO-*n*C_5_H_11_)FKKLQ) | 6.74 | 91 | |  | | 684.9763 [M+2H]^2+^ | 684.9737 | |
| **BPC596** | c(KKLKK(LK-CO-*n*C_5_H_11_)FKKLQ) | 7.01 | 98 | |  | | 805.5658 [M+2H]^2+^ | 805.5646 | |
| **BPC592** | c(KKLKK(CO-C_6_H_13_)FKKLQ) | 7.04 | 91 | |  | | 705.9998 [M+2H]^2+^ | 705.9997 | |
| **BPC594** | c(KKLKK(CO-C_8_H_17_)FKKLQ) | 7.61 | 86 | |  | | 691.9841 [M+2H]^2+^ | 691.9836 | |
| **BPC530** | c(KKLKK(CO-C_11_H_23_)FKKLQ) | 8.01 | 83 | |  | | 727.0233 [M+2H]^2+^ | 727.0237 | |
| **BPC524** | c(KKLKK(CO-C_11_H_22_OH)FKKLQ) | 8.04 | 82 | |  | | 735.0207 [M+2H]^2+^ | 735.0226 | |
| **BPC502** | c(KKLKK(CO-C_15_H_31_)FKKLQ) | 7.78 | 85 | |  | | 755.0546 [M+2H]^2+^ | 755.0555 | |
| **BPC622** | c(KKLKK(CO-C_17_H_33_)FKKLQ) | -^d^ | -^d^ | |  | | 768.0624 [M+2H]^2+^ | 768.0651 | |
| **BPC582** | c(KKLKKFKK(CO-*n*C_5_H_11_)LQ) | 6.88 | 99 | |  | | 684.9763 [M+2H]^2+^ | 684.9761 | |
| **BPC584** | c(KKLKKFK(CO-*n*C_5_H_11_)KLQ) | 6.97 | 98 | |  | | 684.9763 [M+2H]^2+^ | 684.9800 | |
| **BPC586** | c(KKLK(CO-*n*C_5_H_11_)KFKKLQ) | 6.86 | 99 | |  | | 684.9763 [M+2H]^2+^ | 684.9729 | |
| **BPC588** | c(KK(CO-*n*C_5_H_11_)LKKFKKLQ) | 6.89 | 92 | |  | | 684.9763 [M+2H]^2+^ | 684.9744 | |
| **BPC708** | c(KK(CO-C_3_H_7_)LKKFKKLQ) | 6.39 | 99 | |  | | 670.9607 [M+2H]^2+^ | 670.9613 | |
| **BPC590** | c(K(CO-*n*C_5_H_11_)KLKKFKKLQ) | 6.78 | 99 | |  | | 684.9763 [M+2H]^2+^ | 684.9759 | |
| **BPC710** | c(K(CO-C_3_H_7_)KLKKFKKLQ) | 6.57 | 95 | |  | | 670.9607 [M+2H]^2+^ | 670.9586 | |

^a^ CO-C_3_H_7_, butanoyl; CO-C_4_H_9_, pentanoyl; CO-*n*C_5_H_11_, hexanoyl; CO-*iso*C_5_H_11_, 4-methylpentanoyl; CO-C_6_H_13_, 2-methylhexanoyl; CO-C_7_H_15_, octanoyl; CO-C_8_H_17_, 4-methyloctanoyl; CO-C_11_H_22_OH, 12-hydroxylauroyl; CO-C_11_H_23_, lauroyl; CO-C_15_H_31_, palmitoyl; CO-C_17_H_33_, oleoyl.

^b^ HPLC retention time.

^c^ Percentage determined by HPLC at 220 nm from the crude reaction mixture.

^d^ Not observed by HPLC.

**TableB. Sequences, retention times and purities on HPLC, and mass spectrometry data of cyclolipopeptides**

| Peptide | Structure^a^ | *t*_R_ (min)^b^ | Purity (%)^c^ |  | | HRMS (ESI) | |
| --- | --- | --- | --- | --- | --- | --- | --- |
|  |  |  |  | |  | Calculated for | Found |
| **BPC712** | c(KKLKK(CO-C_3_H_7_)fKKLQ) | 6.29 | 97 | |  | 670.9607 [M+2H]^2+^ | 670.9601 |
| **BPC726** | c(KKLKK(LK-CO-C_3_H_7_)fKKLQ) | 6.94 | 97 | |  | 791.5502 [M+2H]^2+^ | 791.5507 |
| **BPC624** | c(KKLKK(CO-*n*C_5_H_11_)fKKLQ) | 6.46 | 99 | |  | 684.9763 [M+2H]^2+^ | 684.9763 |
| **BPC626** | c(KKLKK(CO-*iso*C_5_H_11_)fKKLQ) | 6.45 | 96 | |  | 684.9763 [M+2H]^2+^ | 684.9784 |
| **BPC674** | c(KKLKK(LK-CO-*n*C_5_H_11_)fKKLQ) | 6.39 | 92 | |  | 805.5658 [M+2H]^2+^ | 805.5663 |
| **BPC668** | c(KKLKK(CO-C_7_H_15_)fKKLQ) | 6.78 | 86 | |  | 698.9920 [M+2H]^2+^ | 698.9907 |
| **BPC714** | c(KK(CO-C_3_H_7_)LKKfKKLQ) | 6.21 | 99 | |  | 670.9607 [M+2H]^2+^ | 670.9625 |
| **BPC680** | c(KK(CO-*n*C_5_H_11_)LKKfKKLQ) | 6.49 | 99 | |  | 684.9763 [M+2H]^2+^ | 684.9754 |
| **BPC716** | c(K(CO-C_3_H_7_)KLKKfKKLQ) | 6.08 | 98 | |  | 670.9607 [M+2H]^2+^ | 670.9627 |
| **BPC686** | c(K(CO-*n*C_5_H_11_)KLKKfKKLQ) | 6.09 | 89 | |  | 684.9763 [M+2H]^2+^ | 684.9763 |
| **BPC702** | c(KKLKk(CO-C_3_H_7_)FKKLQ) | 6.65 | 99 | |  | 670.9607 [M+2H]^2+^ | 670.9588 |
| **BPC724** | c(KKLKk(LK-CO-C_3_H_7_)FKKLQ) | 6.97 | 97 | |  | 791.5502 [M+2H]^2+^ | 791.5473 |
| **BPC628** | c(KKLKk(CO-*n*C_5_H_11_)FKKLQ) | 6.40 | 91 | |  | 684.9763 [M+2H]^2+^ | 684.9787 |
| **BPC630** | c(KKLKk(CO-*iso*C_5_H_11_)FKKLQ) | 6.39 | 95 | |  | 684.9763 [M+2H]^2+^ | 684.9786 |
| **BPC672** | c(KKLKk(LK-CO-*n*C_5_H_11_)FKKLQ) | 6.65 | 95 | |  | 805.5658 [M+2H]^2+^ | 805.5667 |
| **BPC666** | c(KKLKk(CO-C_7_H_15_)FKKLQ) | 6.78 | 99 | |  | 698.9920 [M+2H]^2+^ | 698.9905 |
| **BPC678** | c(Kk(CO-*n*C_5_H_11_)LKKFKKLQ) | 6.44 | 94 | |  | 684.9763 [M+2H]^2+^ | 684.9743 |
| **BPC704** | c(Kk(CO-C_3_H_7_)LKKFKKLQ) | 6.25 | 97 | |  | 670.9607 [M+2H]^2+^ | 670.9603 |
| **BPC684** | c(k(CO-*n*C_5_H_11_)KLKKFKKLQ) | 6.50 | 97 | |  | 684.9763 [M+2H]^2+^ | 684.9752 |
| **BPC706** | c(k(CO-C_3_H_7_)KLKKFKKLQ) | 6.82 | 89 | |  | 670.9607 [M+2H]^2+^ | 670.9627 |
| **BPC632** | c(KKLKk(CO-*n*C_5_H_11_)fKKLQ) | 6.36 | 93 | |  | 684.9763 [M+2H]^2+^ | 684.9756 |
| **BPC634** | c(KKLKk(CO-*iso*C_5_H_11_)fKKLQ) | 6.34 | 93 | |  | 684.9763 [M+2H]^2+^ | 684.9778 |
| **BPC718** | c(KKLKK(CO-C_3_H_7_)HKKLQ) | 6.03 | 88 | |  | 665.9559 [M+2H]^2+^ | 665.9571 |
| **BPC728** | c(KKLKK(LK-CO-C_3_H_7_)HKKLQ) | 6.84 | 99 | |  | 786.5454 [M+2H]^2+^ | 786.5482 |
| **BPC636** | c(KKLKK(CO-*n*C_5_H_11_)HKKLQ) | 5.89 | 89 | |  | 679.9716 [M+2H]^2+^ | 679.9720 |
| **BPC638** | c(KKLKK(CO-*iso*C_5_H_11_)HKKLQ) | 5.86 | 90 | |  | 679.9716 [M+2H]^2+^ | 679.9732 |
| **BPC676** | c(KKLKK(LK-CO-*n*C_5_H_11_)HKKLQ) | 6.06 | 91 | |  | 800.5611 [M+2H]^2+^ | 800.5606 |
| **BPC670** | c(KKLKK(CO-C_7_H_15_)HKKLQ) | 6.40 | 87 | |  | 693.9872 [M+2H]^2+^ | 693.9899 |
| **BPC682** | c(KK(CO-*n*C_5_H_11_)LKKHKKLQ) | 6.06 | 96 | |  | 679.9716 [M+2H]^2+^ | 679.9729 |
| **BPC720** | c(KK(CO-C_3_H_7_)LKKHKKLQ) | 6.15 | 89 | |  | 665.9559 [M+2H]^2+^ | 665.9566 |
| **BPC688** | c(K(CO-*n*C_5_H_11_)KLKKHKKLQ) | 6.19 | 91 | |  | 679.9716 [M+2H]^2+^ | 679.9722 |
| **BPC722** | c(K(CO-C_3_H_7_)KLKKHKKLQ) | 5.95 | 88 | |  | 665.9559 [M+2H]^2+^ | 665.9570 |

^a^ CO-C_3_H_7_, butanoyl; CO-*n*C_5_H_11_, hexanoyl; CO-*iso*C_5_H_11_, 4-methylpentanoyl; CO-C_7_H_15_, octanoyl.

^b^ HPLC retention time.

^c^ Percentage determined by HPLC at 220 nm from the crude reaction mixture.

**TableC. Antimicrobial (MIC) and hemolytic activities of cyclolipopeptides containing all L-amino acids^a^**

| Peptide | Structure^b^ | MIC (µM) | | | |  | | Hemolysis (%)^c^ | | | |
| --- | --- | --- | --- | --- | --- | --- | --- | --- | --- | --- | --- |
|  |  | *Xav*^d^ | *Pss*^d^ | *Ea*^d^ | *Fo*^d^ | |  | | 150 μM | 250 μM |  |
| **BPC194** | c(KKLKKFKKLQ) | 3.1-6.2 | 3.1-6.2 | 6.2-12.5 | <3.1 | |  | | 4 ± 1 | 6 ± 1 |  |
| **BPC498** | c(KKLKK(CO-C_7_H_15_)FKKLQ) | 6.2-12.5 | 12.5-25 | 25-50 | 3.1-6.2 | |  | | 78 ± 4 | 100 ± 12 |  |
| **BPC500** | c(KKLKK(CO-C_3_H_7_)FKKLQ) | <3.1 | 6.2-12.5 | 12.5-25 | <3.1 | |  | | 28 ± 5 | 38 ± 4 |  |
| **BPC526** | c(KKLKK(CO-C_4_H_9_)FKKLQ) | <3.1 | 12.5-25 | 12.5-25 | 6.2-12.5 | |  | | 62 ± 2 | 78 ± 3 |  |
| **BPC504** | c(KKLKK(CO-*iso*C_5_H_11_)FKKLQ) | *3.1-6.2* | 6.2-12.5 | 12.5-25 | 3.1-6.2 | |  | | 89 ± 2 | 93 ± 3 |  |
| **BPC528** | c(KKLKK(CO-*n*C_5_H_11_)FKKLQ) | *3.1-6.2* | 6.2-12.5 | 12.5-25 | 6.2-12.5 | |  | | 98 ± 7 | 100 ± 1 |  |
| **BPC596** | c(KKLKK(LK-CO-*n*C_5_H_11_)FKKLQ) | <3.1 | 6.2-12.5 | 12.5-25 | 12.5-25 | |  | | 93 ± 5 | 100 ± 11 |  |
| **BPC592** | c(KKLKK(CO-C_6_H_13_)FKKLQ) | 6.2-12.5 | 12.5-25 | 25-50 | 12.5-25 | |  | | 100 ± 4 | 98 ± 6 |  |
| **BPC594** | c(KKLKK(CO-C_8_H_17_)FKKLQ) | *3.1-6.2* | 6.2-12.5 | 25-50 | 6.2-12.5 | |  | | 93 ± 11 | 96 ± 3 |  |
| **BPC530** | c(KKLKK(CO-C_11_H_23_)FKKLQ) | 6.2-12.5 | 12.5-25 | 12.5-25 | 6.2-12.5 | |  | | 89 ± 7 | 91 ± 9 |  |
| **BPC524** | c(KKLKK(CO-C_11_H_22_OH)FKKLQ) | 6.2-12.5 | 12.5-25 | 25-50 | 12.5-25 | |  | | 100 ± 8 | 100 ± 6 |  |
| **BPC502** | c(KKLKK(CO-C_15_H_31_)FKKLQ) | >50 | >50 | >50 | >50 | |  | | 78 ± 1 | 99 ± 2 |  |
| **BPC622** | c(KKLKK(CO-C_17_H_33_)FKKLQ) | *3.1-6.2* | 25-50 | >50 | >50 | |  | | 91 ± 2 | 97 ± 3 |  |
| **BPC582** | c(KKLKKFKK(CO-*n*C_5_H_11_)LQ) | 6.2-12.5 | 6.2-12.5 | 12.5-25 | 12.5-25 | |  | | 92 ± 3 | 96 ± 10 |  |
| **BPC584** | c(KKLKKFK(CO-*n*C_5_H_11_)KLQ) | 6.2-12.5 | 12.5-25 | 25-50 | 25-50 | |  | | 72 ± 5 | 97 ± 12 |  |
| **BPC586** | c(KKLK(CO-*n*C_5_H_11_)KFKKLQ) | 6.2-12.5 | 6.2-12.5 | 12.5-25 | 12.5-25 | |  | | 92 ± 9 | 95 ± 20 |  |
| **BPC588** | c(KK(CO-*n*C_5_H_11_)LKKFKKLQ) | *3.1-6.2* | 6.2-12.5 | 12.5-25 | 6.2-12.5 | |  | | 95 ± 4 | 92 ± 12 |  |
| **BPC708** | c(KK(CO-C_3_H_7_)LKKFKKLQ) | 6.2-12.5 | 12.5-25 | 25-50 | 3.1-6.2 | |  | | 2 ± 0.5 | 3 ± 1 |  |
| **BPC590** | c(K(CO-*n*C_5_H_11_)KLKKFKKLQ) | *3.1-6.2* | 6.2-12.5 | *6.2-12.5* | 12.5-25 | |  | | 100 ± 9 | 100 ± 9 |  |
| **BPC710** | c(K(CO-C_3_H_7_)KLKKFKKLQ) | *3.1-6.2* | 12.5-25 | 25-50 | *<3.1* | |  | | 7 ± 1 | 13 ± 2 |  |

^a^ Italic values represent the same MIC than that of **BPC194** whereas underlined values correspond to an improvement of the activity compared to **BPC194**.

^b^ CO-C_3_H_7_, butanoyl; CO-C_4_H_9_, pentanoyl; CO-*n*C_5_H_11_, hexanoyl; CO-*iso*C_5_H_11_, 4-methylpentanoyl; CO-C_6_H_13_, 2-methylhexanoyl; CO-C_7_H_15_, octanoyl; CO-C_8_H_17_, 4-methyloctanoyl; CO-C_11_H_22_OH, 12-hydroxylauroyl; CO-C_11_H_23_, lauroyl; CO-C_15_H_31_, palmitoyl; CO-C_17_H_33_, oleoyl.

^c^ Percent hemolysis at 150 and 250 µM plus confidence interval (α = 0.05).

^d^ *Xav*, *Xanthomonas axonopodis* pv. *vesicatoria*; *Pss*, *Pseudomonas syringae* pv. *syringae; Ea*, *Erwinia amylovora; Fo, Fusarium oxysporum.*

**TableD. Antimicrobial (MIC) and hemolytic activities of cyclolipopeptides containing D-amino acids or a histidine residue^a^**

| Peptide | Structure^b^ | MIC (µM) | | | |  | | Hemolysis (%)^c^ | | | |
| --- | --- | --- | --- | --- | --- | --- | --- | --- | --- | --- | --- |
|  |  | *Xav*^d^ | *Pss*^d^ | *Ea*^d^ | *Fo*^d^ | |  | | 150 μM | 250 μM |  |
| **BPC712** | c(KKLKK(CO-C_3_H_7_)fKKLQ) | 3.1-6.2 | 12.5-25 | 25-50 | 3.1-6.2 | |  | | *30* ± 4 | *38* ± 7 |  |
| **BPC726** | c(KKLKK(LK-CO-C_3_H_7_)fKKLQ) | 6.2-12.5 | 12.5-25 | 25-50 | 3.1-6.2 | |  | | 0 ± 1 | 4 ± 2 |  |
| **BPC624** | c(KKLKK(CO-*n*C_5_H_11_)fKKLQ) | 6.2-12.5 | 12.5-25 | 25-50 | *6.2-12.5* | |  | | 53 ± 5 | 64 ± 9 |  |
| **BPC626** | c(KKLKK(CO-*iso*C_5_H_11_)fKKLQ) | 6.2-12.5 | 12.5-25 | 25-50 | 6.2-12.5 | |  | | 51 ± 7 | 54 ± 4 |  |
| **BPC674** | c(KKLKK(LK-CO-*n*C_5_H_11_)fKKLQ) | 6.2-12.5 | *6.2-12.5* | 25-50 | 3.1-6.2 | |  | | 51 ± 2 | 56 ± 2 |  |
| **BPC668** | c(KKLKK(CO-C_7_H_15_)fKKLQ) | *6.2-12.5* | *12.5-25* | *25-50* | 6.2-12.5 | |  | | *79* ± 2 | 79 ± 1 |  |
| **BPC714** | c(KK(CO-C_3_H_7_)LKKfKKLQ) | 12.5-25 | 25-50 | *25-50* | 6.2-12.5 | |  | | *3* ± 1 | *3* ± 0.5 |  |
| **BPC680** | c(KK(CO-*n*C_5_H_11_)LKKfKKLQ) | 12.5-25 | 25-50 | 25-50 | 3.1-6.2 | |  | | 77 ± 2 | 81 ± 4 |  |
| **BPC716** | c(K(CO-C_3_H_7_)KLKKfKKLQ) | 25-50 | *12.5-25* | *25-50* | 3.1-6.2 | |  | | 2 ± 1 | 4 ± 0.5 |  |
| **BPC686** | c(K(CO-*n*C_5_H_11_)KLKKfKKLQ) | 6.2-12.5 | 12.5-25 | 25-50 | *12.5-25* | |  | | 10 ± 0.2 | 14 ± 0.4 |  |
| **BPC702** | c(KKLKk(CO-C_3_H_7_)FKKLQ) | 6.2-12.5 | *6.2-12.5* | 25-50 | 3.1-6.2 | |  | | 1 ± 0.1 | 2 ± 0.3 |  |
| **BPC724** | c(KKLKk(LK-CO-C_3_H_7_)FKKLQ) | 6.2-12.5 | 12.5-25 | 25-50 | <3.1 | |  | | 3 ± 2 | 3 ± 0.5 |  |
| **BPC628** | c(KKLKk(CO-*n*C_5_H_11_)FKKLQ) | 6.2-12.5 | 12.5-25 | 25-50 | *6.2-12.5* | |  | | 19 ± 2 | 34 ± 3 |  |
| **BPC630** | c(KKLKk(CO-*iso*C_5_H_11_)FKKLQ) | 6.2-12.5 | *6.2-12.5* | 25-50 | 6.2-12.5 | |  | | 22 ± 2 | 28 ± 3 |  |
| **BPC672** | c(KKLKk(LK-CO-*n*C_5_H_11_)FKKLQ) | 6.2-12.5 | *6.2-12.5* | 25-50 | 3.1-6.2 | |  | | 33 ± 2 | 33 ± 2 |  |
| **BPC666** | c(KKLKk(CO-C_7_H_15_)FKKLQ) | 12.5-25 | *12.5-25* | *25-50* | 6.2-12.5 | |  | | 61 ± 2 | 73 ± 3 |  |
| **BPC678** | c(Kk(CO-*n*C_5_H_11_)LKKFKKLQ) | 6.2-12.5 | 12.5-25 | 25-50 | 3.1-6.2 | |  | | 68 ± 3 | 73 ± 4 |  |
| **BPC704** | c(Kk(CO-C_3_H_7_)LKKFKKLQ) | *6.2-12.5* | 6.2-12.5 | *25-50* | 12.5-25 | |  | | 9 ± 1 | 16 ± 2 |  |
| **BPC684** | c(k(CO-*n*C_5_H_11_)KLKKFKKLQ) | 6.2-12.5 | 12.5-25 | 25-50 | 6.2-12.5 | |  | | 8 ± 1 | 13 ± 1 |  |
| **BPC706** | c(k(CO-C_3_H_7_)KLKKFKKLQ) | 6.2-12.5 | 6.2-12.5 | *25-50* | 3.1-6.2 | |  | | 35 ± 3 | 46 ± 3 |  |
| **BPC632** | c(KKLKk(CO-*n*C_5_H_11_)fKKLQ) | 25-50 | 12.5-25 | 25-50 | 12.5-25 | |  | | 21 ± 13 | 22 ± 10 |  |
| **BPC634** | c(KKLKk(CO-*iso*C_5_H_11_)fKKLQ) | 25-50 | 12.5-25 | >50 | 6.2-12.5 | |  | | 7 ± 1 | 17 ± 8 |  |
| **BPC718** | c(KKLKK(CO-C_3_H_7_)HKKLQ) | 12.5-25 | 12.5-25 | >50 | 6.2-12.5 | |  | | 3 ± 1 | 4 ± 1 |  |
| **BPC728** | c(KKLKK(LK-CO-C_3_H_7_)HKKLQ) | 6.2-12.5 | 12.5-25 | >50 | <3.1 | |  | | 0 | 7 ± 1 |  |
| **BPC636** | c(KKLKK(CO-*n*C_5_H_11_)HKKLQ) | 12.5-25 | *6.2-12.5* | 25-50 | *6.2-12.5* | |  | | 5 ± 2 | 16 ± 5 |  |
| **BPC638** | c(KKLKK(CO-*iso*C_5_H_11_)HKKLQ) | 25-50 | *6.2-12.5* | 25-50 | 6.2-12.5 | |  | | 6 ± 2 | 8 ± 5 |  |
| **BPC676** | c(KKLKK(LK-CO-*n*C_5_H_11_)HKKLQ) | 6.2-12.5 | 12.5-25 | 25-50 | 3.1-6.2 | |  | | 3 ± 0.3 | 3 ± 1 |  |
| **BPC670** | c(KKLKK(CO-C_7_H_15_)HKKLQ) | 12.5-25 | *12.5-25* | *25-50* | *3.1-6.2* | |  | | 34 ± 1 | 40 ± 1 |  |
| **BPC682** | c(KK(CO-*n*C_5_H_11_)LKKHKKLQ) | 12.5-25 | 12.5-25 | 25-50 | 3.1-6.2 | |  | | 20 ± 3 | 27 ± 1 |  |
| **BPC720** | c(KK(CO-C_3_H_7_)LKKHKKLQ) | 12.5-25 | *12.5-25* | >50 | <3.1 | |  | | *3* ± 1 | *4* ± 1 |  |
| **BPC688** | c(K(CO-*n*C_5_H_11_)KLKKHKKLQ) | 25-50 | 25-50 | >50 | *12.5-25* | |  | | 1 ± 2 | 1 ± 0.2 |  |
| **BPC722** | c(K(CO-C_3_H_7_)KLKKHKKLQ) | 25-50 | >50 | >50 | 3.1-6.2 | |  | | 3 ± 0.2 | 3 ± 0.1 |  |

^a^ Italic values represent the same MIC than that of the corresponding parent peptide in Table S3. Underlined values correspond to an improvement of the activity compared to the corresponding parent peptide in Table S3.

^b^ CO-C_3_H_7_, butanoyl; CO-*n*C_5_H_11_, hexanoyl; CO-*iso*C_5_H_11_, 4-methylpentanoyl; CO-C_7_H_15_, octanoyl.

^c^ Percent hemolysis at 150 and 250 µM plus confidence interval (α = 0.05).

^d^ *Xav*, *Xanthomonas axonopodis* pv. *vesicatoria*; *Pss*, *Pseudomonas syringae* pv. *syringae; Ea*, *Erwinia amylovora; Fo, Fusarium oxysporum.*
